# Elimination of hepatitis C in a hospital characterized by infectious diseases

**DOI:** 10.3389/fpubh.2023.1093578

**Published:** 2023-03-16

**Authors:** Ying Han, Mei Zheng, Huan Meng, Jinyu Han, Jin Chen, Yajie Wang

**Affiliations:** Department of Clinical Laboratory, Beijing Ditan Hospital, Capital Medical University, Beijing, China

**Keywords:** hepatitis C virus, HCV antibody, HCV RNA, screening rules, care cascade

## Abstract

**Background:**

The World Health Organization has proposed to eliminate hepatitis C by 2030, yet there is still a large gap to the goal. Screening for hepatitis C is cost-effective and efficient in medical institutions. The aim of this study was to identify the key populations for HCV antibody screening in hospital characterized by infectious diseases, and provide estimates of the proportion of HCV-infected persons in the Beijing Ditan hospital completing each step along a proposed HCV treatment cascade.

**Methods:**

A total of 105,112 patients who underwent HCV antibody testing in Beijing Ditan hospital between 2017 and 2020 were included in this study. HCV antibody and HCV RNA positivity rate were calculated and compared by chi-square test.

**Results:**

The positivity rate of HCV antibody was 6.78%. The HCV antibody positivity rate and the proportion of positive patients showed an upward trend along with age in the five groups between 10–59 years. In the contrary, a decreasing trend was observed in the three groups above 60 years. Patients with positive HCV antibody were mainly from the Liver Disease Center (36.53%), the Department of Integrative Medicine (16.10%), the Department of Infectious Diseases (15.93%) and the Department of Obstetrics and Gynecology (9.44%). Among HCV antibody positive patients, 6,129 (85.95%) underwent further HCV RNA testing, of whom 2097 were HCV RNA positive, the positivity rate was 34.21%. Of the patients who were HCV RNA positive, 64.33% did not continue with HCV RNA testing. The cure rate for HCV antibody positive patients was 64.98%. Besides, there was a significant positive correlation between HCV RNA positivity rate and HCV antibody level (*r* = 0.992, *P* < 0.001). The detection rate of HCV antibody among inpatients showed an upward trend (*Z* = 5.567, *P* < 0.001), while the positivity rate showed a downward trend (*Z* = 2.2926, *P* = 0.0219).

**Conclusions:**

We found that even in hospitals characterized by infectious diseases, a large proportion of patients did not complete each step along a proposed HCV treatment cascade. Besides, we identified key populations for HCV antibody screening, namely: (1) patients over 40 years of age, especially those aged 50–59 years; (2) the Department of Infectious Diseases and the Department of Obstetrics and Gynecology patients. In addition, HCV RNA testing was highly recommended for patients with HCV antibody levels above 8 S/CO.

## 1. Introduction

Hepatitis C is an inflammation of the liver caused by the hepatitis C virus (HCV). This virus can cause both acute and chronic hepatitis, ranging in severity from a mild illness to a serious, lifelong illness including liver cirrhosis and cancer. Hepatitis C has now become a serious public health problem due to its global prevalence. According to the World Health Organization (WHO), ~1.5 million people in 2019 were newly infected with HCV, while 58 million people had chronic HCV infection, exhibiting a 0.8% prevalence rate in the general population. Approximately 290,000 people died from hepatitis C mostly due to cirrhosis and hepatocellular carcinoma (primary liver cancer) (1).

China is known to have a large population, with many patients suffering from infectious diseases. Relevant studies have shown that although the prevalence of HCV is low in China, it is one of the countries with the largest number of HCV-infected patients in the world (2). It was estimated that there were ~10 million cases of HCV infection in China (3). However, from 2002 to 2021, the Chinese Center for Disease Control and Prevention (CDC) has received only a total of about three million reported cases, which makes about 70% HCV-infected people not being found or treated (4). Since HCV-related tests are not included in routine physical examination, and most patients with acute HCV infection have only slight discomfort or are asymptomatic, such patients are often ignored, eventually leading them to suffer from liver cirrhosis or hepatocellular carcinoma. Relevant studies have shown that for the first time, patients with decompensated liver cirrhosis caused by HCV spent on average 43 days in hospital, resulting in a huge medical and economic burden (5). In May 2016, the World Health Assembly adopted the first *Global health sector strategy on viral hepatitis, 2016–2020*, which stated that by 2030, an increase should be observed in the diagnosis of hepatitis C to 90%, the treatment rate should be increased to 80% (6). However, in 2019, only 21% of chronic HCV infections were diagnosed, treatment was given to 25% of patients in the Asia pacific region, which reveals a huge gap from the target (7). A study has projected that antiviral therapy could only prevent <15% of liver-related deaths caused by hepatitis C between 2002 and 2030. In the future, the fundamental way to reduce the number of deaths related to hepatitis C may include diagnosing and treating more patients rather than developing better treatments (8). Multiple studies have shown that screening for hepatitis C is cost-effective and efficient in medical institutions (9, 10). Here, we retrospectively analyzed the data of hepatitis C patients to identified the key populations for HCV antibody screening in hospital characterized by infectious diseases and provide estimates of the proportion of HCV-infected persons in the Beijing Ditan hospital completing each step along a proposed HCV treatment cascade. Our findings aimed to provide useful information for the development of hepatitis C screening strategies in China.

## 2. Materials and methods

### 2.1. Study design and patients

We retrospectively analyzed the data of HCV antibody and HCV RNA tests in Beijing Ditan Hospital from January 2017 to December 2020. Beijing Ditan Hospital is a Grade-A tertiary hospital featuring infectious diseases. It involves a comprehensive range of departments and a widely distributed patient population. The study was conducted in accordance with the *Declaration of Helsinki* and approved by the Ethics Committee of Beijing Ditan Hospital, Capital Medical University. The ethical approval number is NO. DTEC-KY2022-027-01. Patients with incomplete information were excluded from this study and only the first test results of patients were retained. Ultimately, a total of 105,112 patients tested for HCV antibodies were included in this study.

### 2.2. Detection of HCV antibody and HCV RNA

Plasma samples were separated from the whole blood by centrifugation, and 150 μl of plasma was used to detect the HCV antibody by ARCHITECT i4000SR (Abbott, America). A value of S/CO <1 was considered non-reactive, while a value of S/CO ≥1 was considered reactive. The experiments were repeated twice. If both retests were non-reactive, the detection of HCV antibody was determined as negative, but if one or two retests were reactive, then it was considered positive. Six hundred fifty microliter centrifuged plasma was used to detect the presence of HCV RNA, detection was performed on a fully automated Roche COBAS AmpliPrep instrument, directly docked to the Roche COBAS TaqMan 96 Analyzer. All assay procedures were performed according to the manufacturer's protocols.

### 2.3. Statistical analysis

The data were analyzed using SPSS 24.0 and SAS 9.2 statistical software. The measured data were expressed as mean ± standard deviation (SD), while the counting data were expressed as the number of cases (percentage). The Chi-square test was used to compare the positivity rates between different groups. The Cochran-Armitage test was used to evaluate the trend. All *P*-values were based on two-tailed tests, and *P* < 0.05 was considered statistically significant.

## 3. Results

### 3.1. Demographic characteristics of the study population

A total of 105,112 patients tested for HCV antibody in Beijing Ditan hospital from January 2017 to December 2020 were included in this study. Of 105,112 patients, 54,616 were males, and 50,496 were females, with an average age of (43.49 ± 16.21) years. The patients were divided into nine age groups. As shown in Table 1, the main population detected for HCV antibody was 30–39 years group (24.73%), and the least was 10–19 years group (1.62%).

**Table 1:** Demographic characteristics of the included population.

	**Number of patients**	**Percentage (%)**
**Gender**		
Males	54,616	51.96
Females	50,496	48.04
**Age (years)**		
0–9	2,035	1.94
10–19	1,701	1.62
20–29	20,502	19.50
30–39	25,998	24.73
40–49	17,203	16.37
50–59	18,252	17.36
60–69	12,764	12.14
70–79	4,851	4.62
≥80	1,806	1.72
Total	105,112	100

### 3.2. HCV antibody positivity rate in all age groups

**Figure 1:**
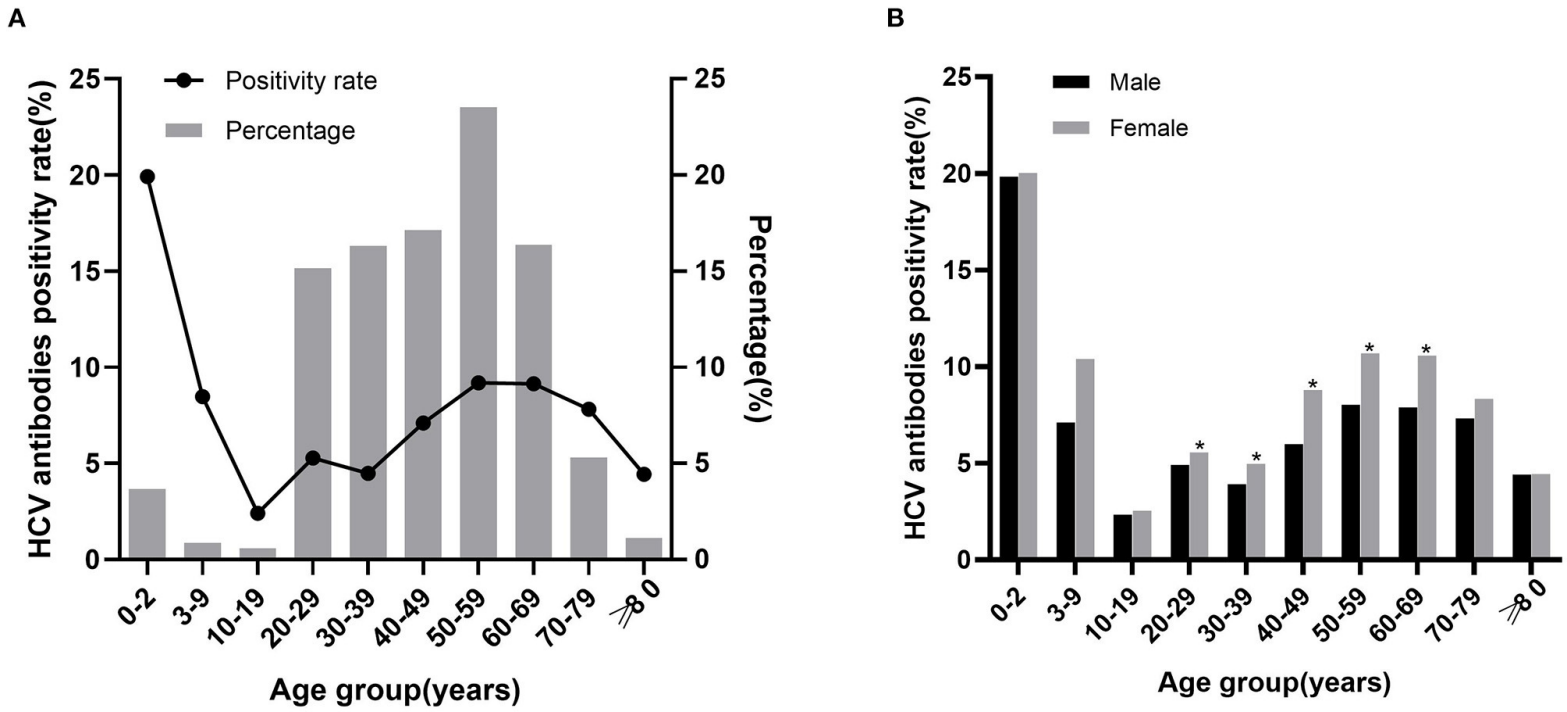
The relationship between HCV antibody positivity rate with age and sex. **(A)** HCV antibody positivity rate in different age groups and age distribution of positive patients. The line chart represents the positivity rate, the histogram represents the percentage of positive patients. **(B)** HCV antibody positivity rate in males and females of different age groups. ^*^P < 0.05.

A total of 7,131 patients were HCV antibody positive, and the positivity rate was 6.78%. The positivity rate of HCV antibody was highest in patients aged 0–2 years (19.92%), followed by 50–59 years (9.19%) and 60–69 years (9.14%). We further analyzed the proportion of positive patients in each group, the largest group was 50–59 years (23.53%), followed by 40–49 years (17.12%). The HCV antibody positivity rate was higher in females (7.52%) than in males (6.11%; *X*^2^ = 82.175, *P* < 0.001) and was significantly different in 20–69 years group (*P* < 0.05) but not in other groups (Figure 1B, Supplementary Table 2). The results demonstrated that, HCV antibody positivity rate and the proportion of positive patients showed an upward trend along with age in the five groups between 10–59 years. In the contrary, a decreasing trend was observed in the three groups above 60 years (Figure 1A, Supplementary Table 1).

### 3.3. HCV antibody-positive patient's departmental origin

**Figure 2:**
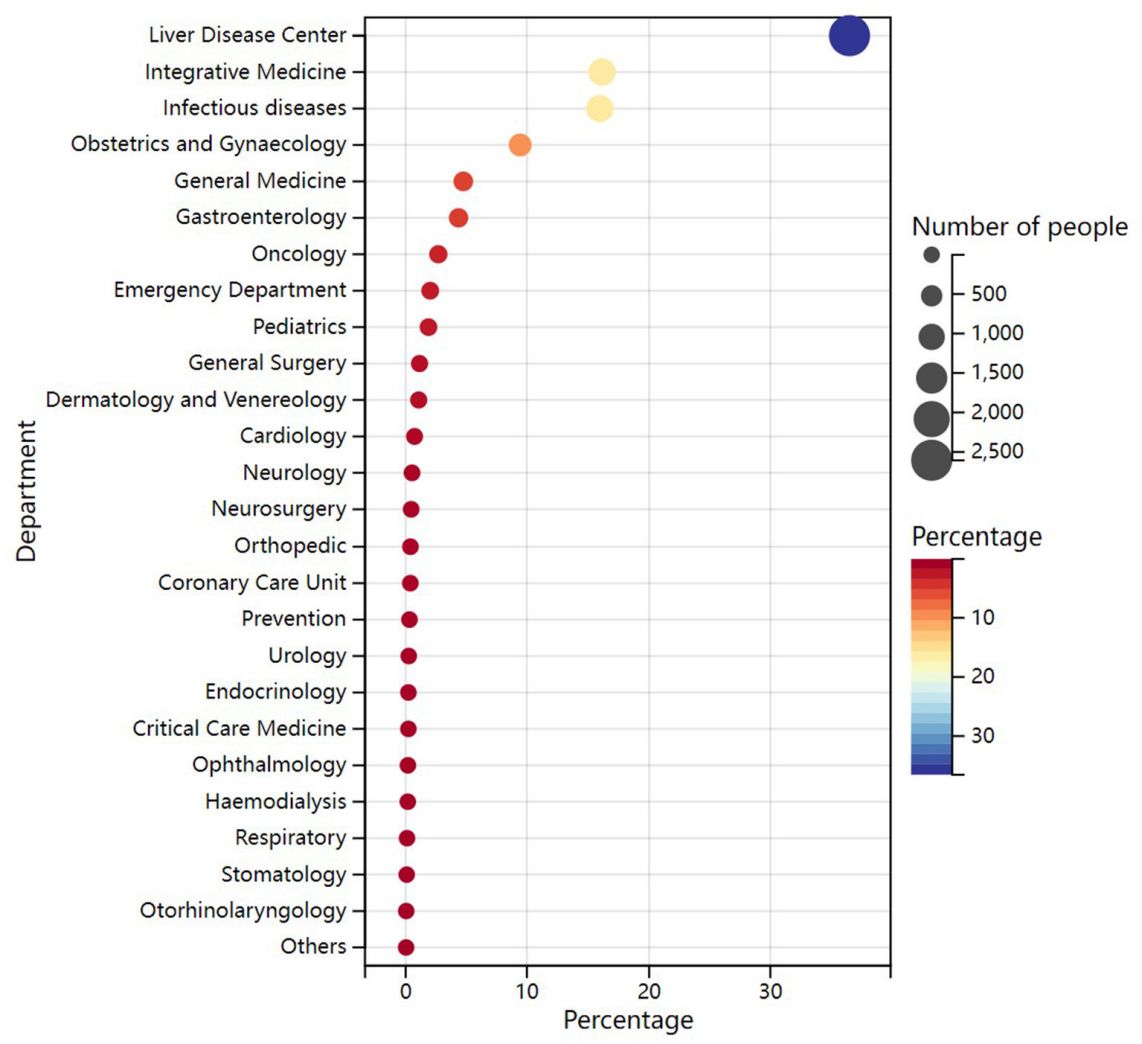
Departmental origin of HCV antibody positive patients. The color of the circle represents the percentage of patients. The size of the circle represents the number of patients, with larger circles indicating a larger number of patients.

To understand the departmental origin of HCV antibody positive patients, we analyzed 7,131 patients from 27 departments. The results demonstrated that patients mainly originated from Liver Disease Center (36.53%), followed by the Department of Integrative Medicine (16.10%), Department of Infectious Diseases (15.93%), and Department of Obstetrics and Gynecology (9.44%; Figure 2, Supplementary Table 3). The results indicated that most of these patients may had a diagnosis of hepatitis C before coming to this hospital. Besides, the department of Obstetrics and gynecology should also be the priority department for hepatitis C screening.

### 3.4. HCV RNA positivity rate in all age groups

**Figure 3:**
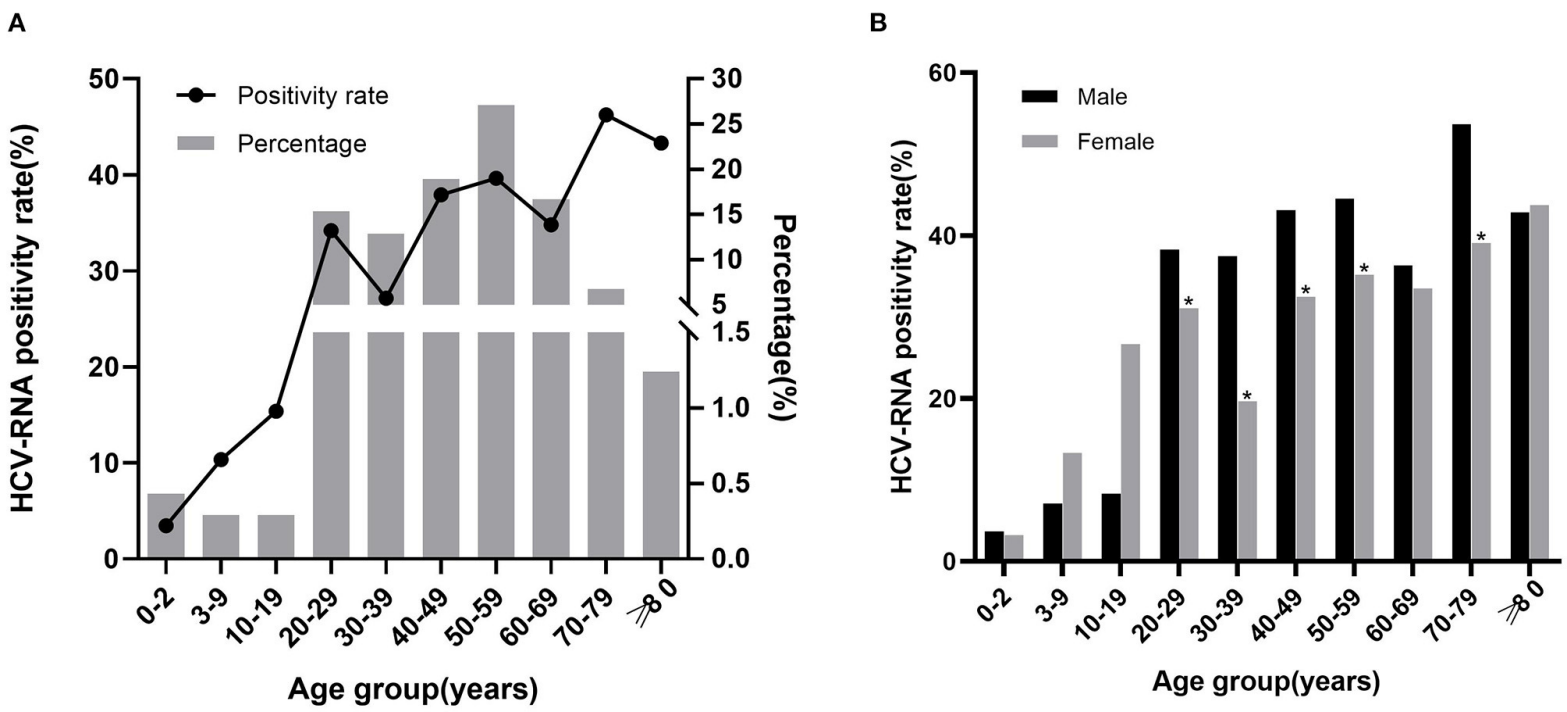
The relationship between HCV RNA positivity rate with age and sex. **(A)** HCV RNA positivity rate in different age groups and age distribution of positive patients. The line chart represents the positivity rate, the histogram represents the percentage of positive patients. **(B)** HCV RNA positivity rate in males and females of different age groups. ^*^P < 0.05.

Of the 7,131 HCV antibody positive patients, 6,129 were tested for HCV RNA. Among 2097 HCV RNA positive patients, the lowest positivity rate was observed in the group of 0–2 years (3.46%), the highest positivity rate was observed in 70–79 years (46.25%). Besides, in the groups above 40 years, the positivity rate was higher than 30%. Overall, the HCV RNA positivity rate increased along with the age. HCV RNA positive patients were mainly concentrated in the age between 20–69, especially in 50–59 years (27.08%; Figure 3A, Supplementary Table 4).

Of the 6,129 patients who underwent HCV RNA testing, 2,844 were males and 3,285 were females. The positivity rate was higher in males (38.92%) than in females (29.92%; *X*^2^ = 54.946, *P* < 0.001), with significant differences in the 20–59 years and 70–79 years (*P* < 0.05) but not in the other groups (Figure 3B, Supplementary Table 5).

### 3.5. Correlation between HCV antibody level and HCV RNA

The correlation between serum HCV antibody levels and HCV RNA positivity rate was evaluated for 7,131 patients with corresponding results. A significant positive correlation between HCV RNA positivity rate and HCV antibody level was found by correlation analysis (r=0.992, *P* < 0.001). Specifically, when HCV antibody level was between 1–3 S/CO, HCV RNA positive patients were rare and the positivity rate was <1%. When the HCV antibody level was between 3 and 8 S/CO, the HCV RNA positivity rate gradually increased to about 20%. When the HCV antibody level was higher than 8 S/CO, the HCV RNA positivity rate was higher than 23.08%. The positive rate reached a maximum of 78% at antibody level between 17 and 18 S/CO (Table 2). The results above indicated the patients were highest recommended to complete HCV RNA test when HCV antibody level was higher than 8 S/CO in clinical practice.

**Table 2:** Correlation between HCV antibody level and HCV RNA.

**HCV antibody level (S/CO)**	**Tested for HCV RNA/HCV antibody (+)**	**HCV RNA test rate (%)**	**HCV RNA (+)/tested for HCV RNA**	**HCV RNA positivity rate (%)**
1–2	1,006/1,204	83.55	7/1,006	0.70
2–3	446/519	85.93	2/446	0.45
3–4	270/296	91.22	7/270	2.59
4–5	181/205	88.29	5/181	2.76
5–6	123/147	83.67	6/123	4.88
6–7	129/142	90.85	12/129	9.30
7–8	120/135	88.89	21/120	17.50
8–9	130/150	86.67	30/130	23.08
9–10	166/190	87.37	49/166	29.52
10–11	212/250	84.80	83/212	39.15
11–12	312/369	84.55	154/312	49.36
12–13	524/593	88.36	265/524	50.57
13–14	778/923	84.29	410/778	52.70
14–15	927/1,067	86.88	530/927	57.17
15–16	542/641	84.56	326/542	60.15
16–17	176/200	88.00	119/176	67.61
17–18	50/60	83.33	39/50	78.00
≥18	37/40	92.50	25/37	67.57

### 3.6. The care cascade of hepatitis C patient

**Figure 4:**
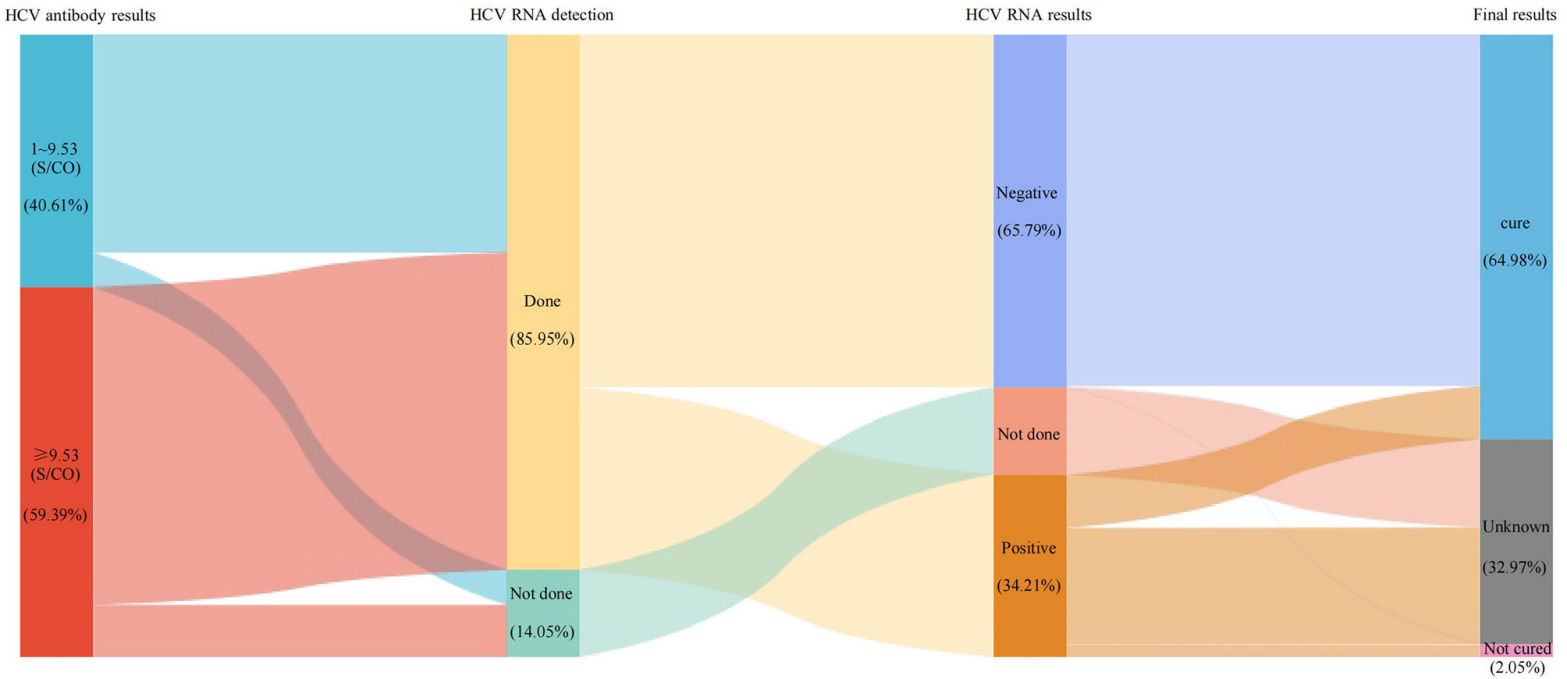
The care cascade of hepatitis C patient. Each column represents a different subgroup. The area between the columns represents the number and percentage of patients. In the first sub-column, 9.53 S/CO represents the mean value of positive HCV antibodies.

To assess the treatment completion rate of hepatitis C patients at this hospital, we further matched HCV antibody and HCV RNA results in 7,131 HCV antibody positive patients over 4 years and conducted a care cascade analysis. Among them, 6,129 (85.95%) underwent further HCV RNA testing, the positivity rate was 34.21% (2,097/6,129). In the proportion of HCV RNA positive patients, there were only 35.67% patients (748/2,097) complete the whole treatment care cascade. Importantly, in the proportion who complete the treatment, 81.42% patients (609/748) reached the treatment endpoint. Besides, we can conclude from the second sub-column (Figure 4), there were even 14.05% HCV antibody positive patients (1,002/7,131) not complete the HCV RNA testing. The results suggested that, even in the hospital characterized by infectious disease, there were still a large portion (64.33%, 1,349/2,097) of patients not complete the treatment.

## 4. Discussion

While the global HCV infection rate is 3%, China's infection rate is about 3.2%, which is slightly higher than the global infection rate. China is also one of the countries with the highest number of HCV infections in the world.

In this study, we compared the positivity rate of HCV antibody among nine age groups. The highest positivity rate of 19.92% was observed in patients aged 0–2 years, which was significantly higher than other groups. There were 230 patients <1 year old in the group of 0–2 years. The reason for the high HCV antibody positive rate in this group may be due to passively acquired HCV antibodies. These antibodies were considered to exist until 18 months after birth (11–13), the HCV RNA test should be carried out in this stage to determine an active infection rather than HCV antibody test (14). Interestingly, only nine patients aged 0–2 years were positive for HCV RNA in this study, with a positive rate of 3.46%. Similar to epidemiological surveys in the USA (15), the HCV antibody positivity rate were lowest in 10–19 years old. The positivity rate of HCV antibody in patients aged 10–59 years showed an increase along with the age, but in patients over 60 years old, it showed a decreased trend with age, which was consistent with previous research (10, 16, 17).

We further analyzed the percentage of HCV antibody positive patients in different age groups. They were mainly concentrated in 20–69 years, particularly in 50–59 years. In this study, both the positivity rate and the number of patients were significantly higher in those over 40 years than in under 40 years (*P* < 0.001), which was consistent with previous studies (18, 19). We speculated that this is primarily due to blood transmission, as HCV antibody testing was not included in the screening routine of blood donation before 1993. Additionally, older patients had less access to learn about the hepatitis C along with the lack of awareness of proactive hepatitis C screening. The positivity rate was found to be low in young people, which has proven the necessity of strict management of blood products and the implementation of hepatitis C prevention policy. The gradual decrease in the positivity rate in patients over 60 years old may be attributed to the death of some patients due to HCV-related cirrhosis or hepatocellular carcinoma (20).

Patients with positive HCV antibody in this hospital mainly originated from the Liver Disease Center and the Department of Integrative Medicine (mainly treats patients with liver disease), as most of the patients were undergoing treatment in this hospital or already knew they had HCV infection. Notably, 15.93% of the patients originated from the Department of Infectious Diseases, suggesting that a larger number of high-risk groups for HCV infection exist in the Department of Infectious Diseases, such as syphilis patients, men who have sex with men, HIV-infected patients and their sexual partners or those with a history of high-risk sexual behavior. Studies have shown that HIV-1 infected men who have sex with men (MSM) have a reinfection rate of 5.27/100 person years after successful treatment for HCV (21). Therefore, screening in patients with diseases that have a similar transmission route to HCV should be emphasized. About 10% of patients also originated from the Department of Obstetrics and Gynecology, studies have reported that the chance of HCV transmission from an HCV antibody positive mother to her newborn was about 2%; if the mother was positive for HCV RNA at the time of delivery, the chance of transmission could be 4% to 7% (3). Therefore, it is necessary to enhance screening for hepatitis C in patients in the Department of Obstetrics and Gynecology. Newborns born to HCV antibody positive mothers should also be tested for HCV antibody on a long-term basis, preferably with HCV RNA testing if possible.

The detection of HCV antibody can only be used as the basis for preliminary screening method, as it may be positive in the case of previous HCV infection, spontaneous virus clearance, or recovery after treatment. HCV RNA was considered as the “gold standard” for the diagnosis of HCV infection and the basis for the effectiveness of antiviral therapy. Previous studies have shown that only 50% of HCV antibody positive patients undergo HCV RNA testing (22), while in this current study was 85.95%, which was higher than the reported. HCV RNA positivity rate was 34.10%, which was lower than other hospitals in China (16), possibly because some patients cleared HCV spontaneously or after treatment. Of the 2,097 patients who were HCV RNA positive, 64.33% did not continue with HCV RNA testing, which may mean that a large number of patients were not receiving regular treatment. Our data showed that there was still a large gap compared to the targets set by the WHO. Besides, regardless of HCV antibody levels, a large proportion of patients were not tested for HCV RNA. Our results indicated that, the HCV antibody levels were strongly correlated with the HCV RNA positivity rate, and the results further indicated when HCV antibody levels were 8–9 S/CO, the HCV RNA positivity rate had exceeded 20%. Therefore, we strongly suggest that all patients with HCV antibody levels higher than 8 S/CO should be tested for HCV RNA to ensure more patients were diagnosed and treated.

The Chinese guidelines for the prevention and treatment of hepatitis C suggest that all patients with positive HCV RNA should be treated irrespective of liver cirrhosis, chronic kidney disease, or extrahepatic manifestations (3). However, only a minority received professional treatment (22). With the availability of direct antiviral agents (DAA), hepatitis C can be cured in only 3 months with fewer side effects. The cure rate in this study was 96.95%. Notably, seven patients had undetectable HCV RNA in their blood during treatment but were detected again in the subsequent treatment, i.e., virological breakthrough. Therefore, HCV RNA testing should be continued even if the patient achieves a sustained virologic response (SVR).

Among HCV antibody positive patients, HCV RNA positivity rate increased along with age, similar to the other studies (16, 19), which may indicate that younger patients were more likely to achieve the cure. It may also indicate that the younger the age, the lower the prevalence and the higher the false positive rate. Patients aged 50–59 years were also the main group of HCV RNA positive patients, accounting for 27.08%. In addition, the positivity rate and the number of patients over 40 years were higher than those under 40 years. This result reaffirms that patients over 40 years were the main population target for HCV infection and treatment, especially those aged 50–59 years.

In this study, the rate of HCV antibody positivity was higher in females than in males (7.52 vs. 6.11%, *P* < 0.001). Further comparison showed that HCV RNA positivity rate was higher in males than in females (38.92 vs. 29.92%, *P* < 0.001). Studies have shown that the best critical value for predicting HCV RNA positivity using HCV antibody was higher in women than in men (16), which may indicate that more women were false positive. Previous studies (16, 17, 20, 23) have shown conflicting conclusions in the comparison results of HCV antibody and HCV RNA positivity rates between men and women. Although the specific reason was not clear, the differences might be related to the different survey populations, different lifestyles, or other reasons.

To our knowledge, this is the early research focused on hepatitis screening and care cascade in a hospital characteristic by infectious diseases in China. The sample size was large, and the source of patients were widely distributed. However, there were some shortcomings in our study. First, our data were collected from mono-centric and the results may not be representative of hospitals in other parts of China. Second, we cannot provide information about the risk factors for HCV infection as this study was designed as a retrospective analysis. In the future, we will conduct a long-term follow-up of HCV-infected patients to further assess the risk factor of patients.

## 5. Conclusion

In conclusion, by analyzing the data from this hospital, we found that even in hospitals characterized by infectious diseases, 14.05% (1,002/7,131) of HCV antibody positive patients still do not undergo HCV RNA testing. Among HCV RNA positive patients, 64.33% did not complete the treatment care cascade, which may mean that a large number of patients were missing the regular treatment. Besides, we identified key populations for HCV antibody screening, namely: (1) patients over 40 years of age, especially those aged 50–59 years; (2) the Department of Infectious Diseases and the Department of Obstetric and gynecological patients. In addition, HCV RNA testing was highly recommended for patients with HCV antibody levels above 8S/CO.

## Data availability statement

The original contributions presented in the study are included in the article/Supplementary material, further inquiries can be directed to the corresponding author.

## Ethics statement

The study was conducted in accordance with the Declaration of Helsinki and approved by the Ethics Committee of Beijing Ditan Hospital, Capital Medical University. The ethical approval number is NO. DTEC-KY2022-027-01.

## Author contributions

Conceiving and designing experiments: YW. Paper writing and revising: YH. Data collection: YH, MZ, and HM. Statistical analysis: YH, JH, and JC. All authors have read and agreed to the published version of the manuscript and given approval to the final version of the manuscript.

## References

[1] World Health Organization. Global Progress Report on HIV, Viral Hepatitis and Sexually Transmitted Infections, 2021: Accountability for the Global Health Sector Strategies 2016–2021: Actions for Impact. Web annex 2. Data methods. Geneva: World Health Organization. (2021). Available online at: https://apps.who.int/iris/handle/10665/341412 (accessed January 03, 2023).

[2] Blach S Zeuzem S Manns M Altraif I Duberg AS Muljono DH. Global prevalence and genotype distribution of hepatitis C virus infection in 2015: a modelling study. Lancet Gastroenterol Hepatol. (2017) 2:161–76. 10.1016/S2468-1253(16)30181-9 28404132

[3] Chinese Chinese Society of Hepatology and Chinese Society of Infectious Diseases Chinese Medical Association. Guidelines for the prevention and treatment of hepatitis C (2019version). Chin J Infect Dis. (2020) 38:9–28. 10.3760/cma.j.issn.1000-6680.2020.01.00430704229

[4] China Liver Health Chinese Chinese Society of Hepatology Chinese Medical Association Chinese Chinese Society of Laboratory Medicine Chinese Medical Association Hospital Hospital Infection Management Committee of Chinese Hospital Association. In-hospital process for viral hepatitis C screening and management in China (Draft). Chin J Hepatol. (2021) 29:319–25. 10.3760/cma.j.cn501113-20210401-00157 33979957 PMC12814419

[5] McDonald SA Innes HA Aspinall EJ Hayes PC Alavi M Valerio H. Inpatient hospital burden of hepatitis C-diagnosed patients with decompensated cirrhosis. Liver Int. (2018) 38:1402–10. 10.1111/liv.13681 29288595

[6] World Health Organization. Global Health Sector Strategy on Viral Hepatitis 2016–2021. Towards Ending Viral Hepatitis. Geneva: World Health Organization. (2016). Available online at: https://apps.who.int/iris/handle/10665/246177 (accessed January 03, 2023).

[7] Le LV Blach S Rewari B Chan P Fuqiang C Ishikawa N. Progress towards achieving viral hepatitis B and C elimination in the Asia and Pacific region: results from modelling and global reporting. Liver Int. (2022) 42:1930–4. 10.1111/liv.15131 34894047

[8] Volk ML Tocco R Saini S Lok AS. Public health impact of antiviral therapy for hepatitis C in the United States. Hepatology. (2009) 50:1750–5. 10.1002/hep.23220 19824079

[9] Orkin C Leach E Flanagan S Wallis E Ruf M Foster G. High prevalence of hepatitis C (HCV) in the emergency department (ED) of a London hospital: should we be screening for HCV in ED attendees? Epidemiol Infect. (2015) 143:2837–40. 10.1017/S0950268815000199 25672420 PMC9151058

[10] Liu L Xu H Hu Y Shang J Jiang J Yu L. Hepatitis C screening in hospitals: find the missing patients. Virol J. (2019) 16:1–9. 10.1186/s12985-019-1157-1 30992019 PMC6469068

[11] Mack CL Gonzalez-Peralta RP Gupta N Leung D Narkewicz MR Roberts EA. NASPGHAN practice guidelines: diagnosis and management of hepatitis C infection in infants, children, and adolescents. J Pediatr Gastroenterol Nutr. (2012) 54:838–55. 10.1097/MPG.0b013e318258328d 22487950

[12] Kesson A. Diagnosis and management of paediatric hepatitis C virus infection. J Paediatr Child Health. (2002) 38:213–8. 10.1046/j.1440-1754.2002.00804.x 12047684

[13] Ceci O Margiotta M Marello F Francavilla R Loizzi P Francavilla A. Vertical transmission of hepatitis C virus in a cohort of 2,447 HIV-seronegative pregnant women: a 24-month prospective study. J Pediatr Gastroenterol Nutr. (2001) 33:570–5. 10.1097/00005176-200111000-00011 11740231

[14] England K Pembrey L Tovo PA Newell ML. Excluding hepatitis C virus (HCV) infection by serology in young infants of HCV-infected mothers. Acta Paediatr. (2005) 94:444–50. 10.1111/j.1651-2227.2005.tb01916.x 16092459

[15] Schillie S Wester C Osborne M Wesolowski L Ryerson AB. CDC recommendations for hepatitis C screening among adults—United States, 2020. MMWR Recomm Rep. (2020) 69:1–17. 10.15585/mmwr.rr6902a1 32271723 PMC7147910

[16] Li Y Zhao L Geng N Zhu W Liu H Bai H. Prevalence and characteristics of hepatitis C virus infection in Shenyang City, Northeast China, and prediction of HCV RNA positivity according to serum anti-HCV level: retrospective review of hospital data. Virol J. (2020) 17:1–8. 10.1186/s12985-020-01316-y 32178702 PMC7077010

[17] Zhou M Li H Ji Y Ma Y Hou F Yuan P. Hepatitis C virus infection in the general population: a large community-based study in Mianyang, West China. Biosci Trends. (2015) 9:97–103. 10.5582/bst.2015.01033 25971694

[18] Chlibek R Smetana J Sosovickova R Gal P Dite P Stepanova V. Prevalence of hepatitis C virus in adult population in the Czech Republic–time for birth cohort screening. PLoS ONE. (2017) 12:e0175525. 10.1371/journal.pone.0175525 28406947 PMC5391198

[19] Zhang M Wu R Xu H Uhanova J Gish R Wen X. Changing incidence of reported viral hepatitis in China from 2004 to 2016: an observational study. BMJ Open. (2019) 9:e028248. 10.1136/bmjopen-2018-028248 31427323 PMC6701656

[20] Lu J Zhou Y Lin X Jiang Y Tian R Zhang Y. General epidemiological parameters of viral hepatitis A, B, C, and E in six regions of China: a cross-sectional study in 2007. PLoS ONE. (2009) 4:e8467. 10.1371/journal.pone.0008467 20041146 PMC2794385

[21] Wan Z Sun P Dzakah EE Huang L Shuai P Liu Y. Reinfection rate of hepatitis C in HIV-1 positive men who have sex with men: a systematic review and meta-analysis. Front Public Health. (2022) 10:855989. 10.3389/fpubh.2022.855989 35968434 PMC9372531

[22] Yehia BR Schranz AJ Umscheid CA Lo Re V III. The treatment cascade for chronic hepatitis C virus infection in the United States: a systematic review and meta-analysis. PLoS ONE. (2014) 9:e101554. 10.1371/journal.pone.0101554 24988388 PMC4079454

[23] Rao HY Sun DG Yang RF Liu F Wang J Feng B. Outcome of hepatitis C virus infection in Chinese paid plasma donors: a 12–19-year cohort study. J Gastroenterol Hepatol. (2012) 27:526–32. 10.1111/j.1440-1746.2011.06880.x 21871021

