# Supplementary material for: Elimination of hepatitis C in a hospital characterized by infectious diseases
Source: Front Public Health. 2023 Mar 16;11:1093578. doi: 10.3389/fpubh.2023.1093578 (PMC10061059; doi:10.3389/fpubh.2023.1093578)
Supplement: Supplementary file 1 [file Data_Sheet_1.PDF]

Supplementary Material

Age group (years)	Positivity rate (%)	Percentage (%)
0-2	19.92	3.67
3-9	8.47	0.86
10-19	2.41	0.57
20-29	5.27	15.15
30-39	4.47	16.30
40-49	7.10	17.12
50-59	9.19	23.53
60-69	9.14	16.37
70-79	7.81	5.31
≥80	4.43	1.12

Supplementary table 1. HCV antibody positivity rate in different age groups and age distribution of positive patients.

Age group (years)	Male positivity rate (%)	Female positivity rate (%)
0-2	19.83	20.03
3-9	7.11	10.40
10-19	2.33	2.54
20-29	4.92	5.56
30-39	3.91	4.97
40-49	5.99	8.80
50-59	8.03	10.69
60-69	7.90	10.58
70-79	7.32	8.33
≥80	4.42	4.44

Supplementary table 2. HCV antibody positivity rate in male and female.

Department	Number of patients	Percentage (%)
Liver Disease Center	2605	36.53
Integrative Medicine	1148	16.10
Infectious diseases	1136	15.93
Obstetrics and Gynaecology	673	9.44
General Medicine	339	4.75
Gastroenterology	311	4.36
Oncology	192	2.69
Emergency Department	144	2.02
Pediatrics	134	1.88
General Surgery	81	1.14
Dermatology and Venereology	76	1.07
Cardiology	52	0.73
Neurology	37	0.52
Neurosurgery	32	0.45
Orthopedic	28	0.39
Coronary Care Unit	27	0.38
Prevention	22	0.31
Urology	18	0.25
Endocrinology	16	0.22
Critical Care Medicine	16	0.22

Ophthalmology	13	0.18
Haemodialysis	12	0.17
Respiratory	8	0.11
Stomatology	6	0.08
Otorhinolaryngology	3	0.04
Others	2	0.03

Supplementary table 3. Departmental origin of HCV antibody positive patients.

Age group (years)	Positivity rate (%)	Percentage (%)
0-2	3.46	0.43
3-9	10.34	0.29
10-19	15.38	0.29
20-29	34.19	15.36
30-39	27.14	12.87
40-49	37.93	18.95
50-59	39.64	27.08
60-69	34.80	16.70
70-79	46.25	6.79
≥ 80	43.33	1.24

Supplementary table 4. HCV RNA positivity rate in different age groups and age distribution of positive patients.

Age group (years)	Male positivity rate (%)	Female positivity rate (%)
0-2	3.68	3.23
3-9	7.14	13.33
10-19	8.33	26.67
20-29	38.31	31.1
30-39	37.50	19.65
40-49	43.15	32.49
50-59	44.54	35.20
60-69	36.38	33.51
70-79	53.64	39.10
≥80	42.86	43.75

Supplementary table 5. HCV RNA positivity rate in male and female.
